# Time-efficient combined morphologic and quantitative joint MRI: an *in situ* study of standardized knee cartilage defects in human cadaveric specimens

**DOI:** 10.1186/s41747-024-00462-0

**Published:** 2024-06-05

**Authors:** Teresa Lemainque, Nicola Pridöhl, Shuo Zhang, Marc Huppertz, Manuel Post, Can Yüksel, Masami Yoneyama, Andreas Prescher, Christiane Kuhl, Daniel Truhn, Sven Nebelung

**Affiliations:** 1 Department of Diagnostic and Interventional Radiology, Medical Faculty, RWTH Aachen University, Pauwelsstr. 30, Aachen, 52074 Germany; 2 Philips GmbH Market DACH, Hamburg, Germany; 3 Philips Japan, Tokyo, Japan; 4 Institute of Molecular and Cellular Anatomy, RWTH Aachen University, Aachen, 52074 Germany

**Keywords:** Cartilage, *In situ* model, Knee joint, Magnetic resonance imaging, Osteoarthritis

## Abstract

**Background:**

Quantitative techniques such as T2 and T1ρ mapping allow evaluating the cartilage and meniscus. We evaluated multi-interleaved X-prepared turbo-spin echo with intuitive relaxometry (MIXTURE) sequences with turbo spin-echo (TSE) contrast and additional parameter maps *versus* reference TSE sequences in an *in situ* model of human cartilage defects.

**Methods:**

Standardized cartilage defects of 8, 5, and 3 mm in diameter were created in the lateral femora of ten human cadaveric knee specimens (81 ± 10 years old; nine males, one female). MIXTURE sequences providing proton density-weighted fat-saturated images and T2 maps or T1-weighted images and T1ρ maps as well as the corresponding two- and three-dimensional TSE reference sequences were acquired before and after defect creation (3-T scanner; knee coil). Defect delineability, bone texture, and cartilage relaxation times were quantified. Appropriate parametric or non-parametric tests were used.

**Results:**

Overall, defect delineability and texture features were not significantly different between the MIXTURE and reference sequences (*p* ≤ 0.47). After defect creation, relaxation times significantly increased in the central femur (T2_pre_ = 51 ± 4 ms [mean ± standard deviation] *versus* T2_post_ = 56 ± 4 ms; *p* = 0.002) and all regions combined (T1ρ_pre_ = 40 ± 4 ms *versus* T1ρ_post_ = 43 ± 4 ms; *p* = 0.004).

**Conclusions:**

MIXTURE permitted time-efficient simultaneous morphologic and quantitative joint assessment based on clinical image contrasts. While providing T2 or T1ρ maps in clinically feasible scan time, morphologic image features, *i.e.*, cartilage defects and bone texture, were comparable between MIXTURE and reference sequences.

**Relevance statement:**

Equally time-efficient and versatile, the MIXTURE sequence platform combines morphologic imaging using familiar contrasts, excellent image correspondence *versus* corresponding reference sequences and quantitative mapping information, thereby increasing the diagnostic value beyond mere morphology.

**Key points:**

• Combined morphologic and quantitative MIXTURE sequences are based on three-dimensional TSE contrasts.

• MIXTURE sequences were studied in an *in situ* human cartilage defect model.

• Morphologic image features, *i.e.*, defect delineabilty and bone texture, were investigated.

• Morphologic image features were similar between MIXTURE and reference sequences.

• MIXTURE allowed time-efficient simultaneous morphologic and quantitative knee joint assessment.

**Graphical Abstract:**

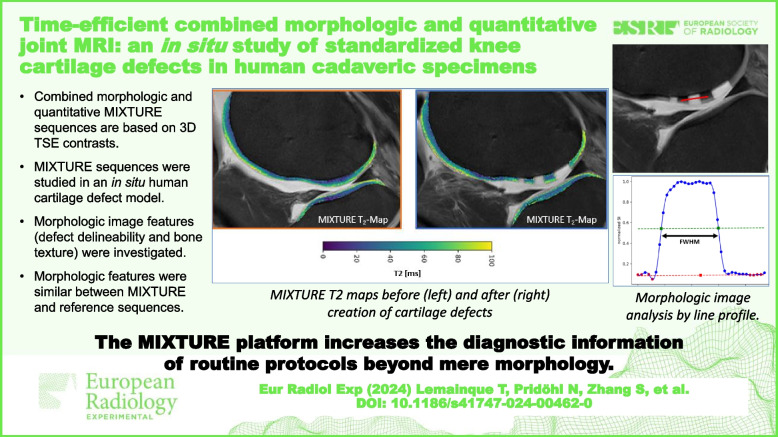

**Supplementary Information:**

The online version contains supplementary material available at 10.1186/s41747-024-00462-0.

## Background

Osteoarthritis is a chronic joint disease with increasing prevalence due to aging and obesity [1]. Magnetic resonance imaging (MRI) is clinically well-suited for diagnosing cartilage degeneration as the hallmark change of osteoarthritis. Traditional MRI sequences such as proton density (PD)-weighted fat-saturated (FS) sequences focus on cartilage morphology, *i.e.*, surface integrity and intratissue signal [2]. They are insensitive to early degenerative changes of tissue morphology, such as partial-thickness defects and fibrillation [3, 4].

Quantitative MRI techniques such as T2 or T1ρ mapping may be beneficial in detecting such changes at a potentially reversible stage [5, 6]. Consensus prevails that adding T2 maps to the routine knee protocol improved sensitivity in detecting cartilage lesions significantly [3, 6]. The literature is less clear on T1ρ: even though the association between cartilage degeneration and prolongation of T1ρ relaxation is well established [7, 8], its potential diagnostic benefits remain to be ascertained. Both mapping techniques have in common that their more widespread adoption is hampered by prohibitively long scan times and other challenges [9, 10].

Combined morphologic and quantitative sequences, such as quantitative double-echo in steady-state, provide morphologic images and T2 maps in clinically feasible scan times [11–13]. Quantitative double-echo in steady-state sequences is diagnostically equivalent to conventional clinical MRI protocols; therefore, they are theorized to (partially) substitute the routine knee protocol while providing additional T2 maps [14]. Nevertheless, the morphologic images lack the clinically familiar contrasts of state-of-the-art turbo spin-echo (TSE) sequences. The “multi-interleaved x-prepared turbo spin-echo with intuitive relaxometry” (MIXTURE) sequence provides an alternative platform for combined imaging [15]. These sequences are designed to acquire at least two morphologic images of different contrast weightings using variable prepulses. Because prepulses, echo times, and spin-lock durations are freely adjustable, the sequences provide quantitative T2 or T1ρ maps as a “by-product” of the morphologic images. In contrast to quantitative double-echo in steady-state, MIXTURE is based on a three-dimensional (3D) TSE acquisition. Preliminary studies have explored its principal clinical applicability, but systematic comparisons with reference sequences and standardized pathologies are lacking [16–20].

This study aimed to evaluate MIXTURE sequences in two principal configurations for their clinical usage and against corresponding two-dimensional (2D) and 3D TSE reference sequences in an *in situ* model of standardized cartilage defects. We hypothesized that the morphologic MIXTURE images are diagnostically on par with their reference sequence counterparts while additionally providing T2 and T1ρ maps in clinically feasible time frames.

## Methods

### Study design

The local Institutional Review Board approved this prospective *in situ* imaging study on human cadaveric knee joint specimens (Ethical Committee, RWTH Aachen University, EK180/16) conducted in 2022 and 2023. Fresh-frozen and nonfixated knee joint specimens from body donors who had given written informed consent prior to study initiation were provided by the local Institute of Anatomy (RWTH Aachen University, Germany). Moderate-to-severe cartilage degeneration of the lateral compartment, such as substantial tissue loss or focal lesions, was screened for during standard clinical scanning (using 2D TSE PD-weighted FS imaging) and defined as an exclusion criterion. Based on a preliminary analysis of the first three specimens, a minimum sample size of 8 was calculated using a statistical power of 80%, a significance level of 0.01, and an effect size (*i.e.*, Cohen’s *d* [21]) of 1.24. Hence, ten knee joint specimens were included.

### Workflow

Specimens were left to thaw at room temperature for 24 h. MRI was performed before and after creating standardized cartilage defects. On day 1, predefect MRI was performed. The specimens were kept at 5 °C overnight. On day 2, cartilage defects were created, and postdefect MRI was performed immediately afterward.

### Cartilage defects

**Fig. 1:**
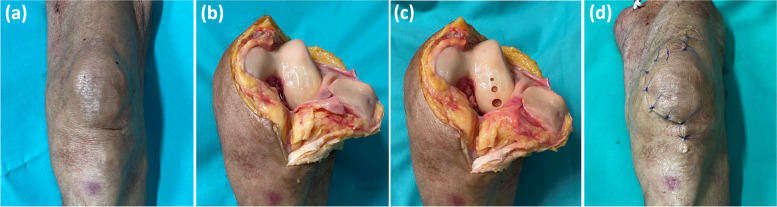
Standardized cartilage defect model. **a** Intact knee joint. **b** Complete surgical exposure of the knee joint specimen through the longitudinal arthrotomy, medial peripatellar incision, and lateral eversion of the patella. **c** By use of biopsy punches, cartilage defects of variable diameters, *i.e.*, 3 mm (top), 5 mm (center), and 8 mm (bottom), were aligned anteroposteriorly. **d** The wound was closed by layer-wise suturing under continuous irrigation

We present in Fig. 1 the standardized step-wise creation of the cartilage defects. N.P. (pregraduation medical student, 2 years of experience) created the defects. First, the knee joint was accessed through a median longitudinal skin incision and a medial peri-patellar approach. Once the joint was flexed, the patella was everted laterally to fully expose the joint. Second, the weight-bearing region of the lateral femoral condyle was identified. Three defects of 3, 5, and 8 mm in diameter were created in the lateral femoral condyle perpendicular to the condyle’s bone contour. The cartilage tissue was removed using skin biopsy punches of corresponding diameters and surgical scalpels. Particular care was taken to maintain the integrity of the subchondral lamella. Third, the joint was thoroughly and continuously irrigated with 0.9% saline solution to remove surgical debris and excess air. Fourth, the joint was sutured layer-wise.

### MRI acquisition

All scans were performed on a 3-T MRI scanner (Elition X, Philips, Best, The Netherlands) using an eight-channel transmit-receive knee coil. Specimens were positioned feet-first, supine, and in approximately 30° of flexion in line with clinical positioning. Two MIXTURE sequences were acquired: PD-weighted FS images with T2 maps (scan time 4:59 min:s) and T1-weighted images with T1ρ maps (scan time 6:30 min:s), followed by the respective reference sequences (2D TSE and 3D TSE). The first MIXTURE sequence was a combination of PD-weighted FS (using a spectral attenuated inversion-recovery [SPAIR] prepulse) and T2-weighted morphologic (using a T2-preparation module of 50 ms) acquisitions, in an interleaved manner. Voxel-wise T2 relaxation times were determined based on the two images by monoexponential fitting, and T2 maps were subsequently reconstructed on the scanner workstation using prototype software. The second MIXTURE sequence was a combination of a T1-weighted (without preparation) and two spin lock-prepared T1ρ-weighted FS morphologic acquisitions (using T1ρ-preparation modules of 25 ms and 50 ms, respectively, and SPAIR prepulses). Voxel-wise, T1ρ relaxation times were determined based on the three images by mono-exponential fitting, and T1ρ maps were reconstructed accordingly. Further details on the MIXTURE sequences can be found in the literature [15]. 2D TSE reference PD-weighted FS and T1-weighted sequences were included per our clinical knee protocol, while 3D TSE reference PD-weighted FS and T1-weighted sequences were obtained from the vendor and included. Table 1 summarizes the sequence parameters.

**Table 1:** MRI sequence parameters

Parameter	MIX 1	2D TSE PD-FS	3D TSE PD-FS	MIX 2	2D TSE T1-weighted	3D TSE T1-weighted
Sequence type	3D TSE	2D TSE	3D TSE	3D TSE	2D TSE	3D TSE
Orientation	Sagittal					
TR [ms]	1,200	3,000	1,100	600	582	400
TE [ms]	N/A	40	N/A	N/A	15	N/A
TE_eff_ [ms]	125	N/A	125	22	N/A	36
TE_equiv_ [ms]	46	N/A	46	13	N/A	21
Echo train length [*n*]	35	11	35	12	5	8
Refocusing pattern	“MSK PD FS”	“No”	“MSK PD FS”	“Spine view T1”	“Constant” (110°)	“MSK T1”
Compressed SENSE factor	4.5	2.5	3.5	6	2	6
NSA [*n*]	1	2	1	1	2	2
Fat saturation	SPAIR—none	SPIR	SPAIR	None—SPAIR	None	None
T2-prep TE [ms]	0, 50	N/A	N/A	N/A	N/A	N/A
SL-prep TSL [ms]	N/A	N/A	N/A	0, 25, 50	N/A	N/A
SL-prep frequency [Hz]	N/A	N/A	N/A	500	N/A	N/A
Scan time [min:s]	4:59	4:06	2:57	6:38	4:23	4:22
FOV [mm^2^]	140 × 140					
Acquisition matrix [px]	304 × 304					
Reconstruction matrix [px]	512 × 512					
Fat shift direction	Anteroposterior					
Phase oversampling [%]	12 + 12	30 + 30	12 + 12	12 + 12	33 + 33	12 + 12
Slices [*n*]	43					
Slice thickness [mm]	3					
Slice oversampling [%]	12	N/A	12	100	N/A	12

Two MIXTURE sequences were acquired, combining morphologic imaging with quantitative mapping, and 2D TSE and 3D TSE reference sequences of the same weighting. PD-weighted FS images were combined with quantitative T2 maps (“MIX 1”) and T1-weighted images with T1ρ maps (“MIX 2”). Note that for 3D TSE sequences, TE_eff_ and TE_equiv_ denote the effective and equivalent TE, respectively, as mediated by the choice of the refocusing pattern. In contrast, the 2D TSE sequence uses a constant refocusing flip angle that a single TE can describe. During the TSE readout, different refocusing patterns with variable order and magnitude of the flip angles are employed as designated by the manufacturer. T2-prep TE and SL-prep TSL refer to the duration of the preparation modules that MIXTURE employs to generate the respective contrast weightings

*FOV* Field of view, *FS* Fat-saturated, *MIXTURE* Multi-interleaved x-prepared turbo spin-echo with intuitive relaxometry, *MSK* Musculoskeletal, *N/A* Not applicable, *NSA* Number of signal averages, *PD* Proton density, *Prep* Preparation, *TE* Echo time, *TE*_*eff*_ Effective TE, *TE*_*equiv*_ Equivalent TE, *TSE* turbo spin-echo, *TSL* Spin lock time, *TR* Repetition time, *SENSE* Sensitivity encoding, *SL* Sin lock, *SPAIR* Spectral attenuated inversion-recovery, *SPIR* Spectral presaturation with inversion-recovery

Notably, as 3D TSE acquisitions, the reference 3D TSE and MIXTURE sequences can, in principle, be acquired at isotropic resolution. In this study, however, we aimed to match the 3D TSE sequences to the 2D reference TSE sequences, *i.e.*, the clinical reference standard, for voxel-to-voxel comparisons. Consequently, the 3D TSE sequences were acquired analogously to the 2D TSE sequence, *i.e.*, using thicker slices and higher in-plane resolution than achievable with isotropic image acquisitions.

### Image analysis

Quantitative analyses were performed in Python version 3.9.9 [22].

#### Defect delineability

**Fig. 2:**
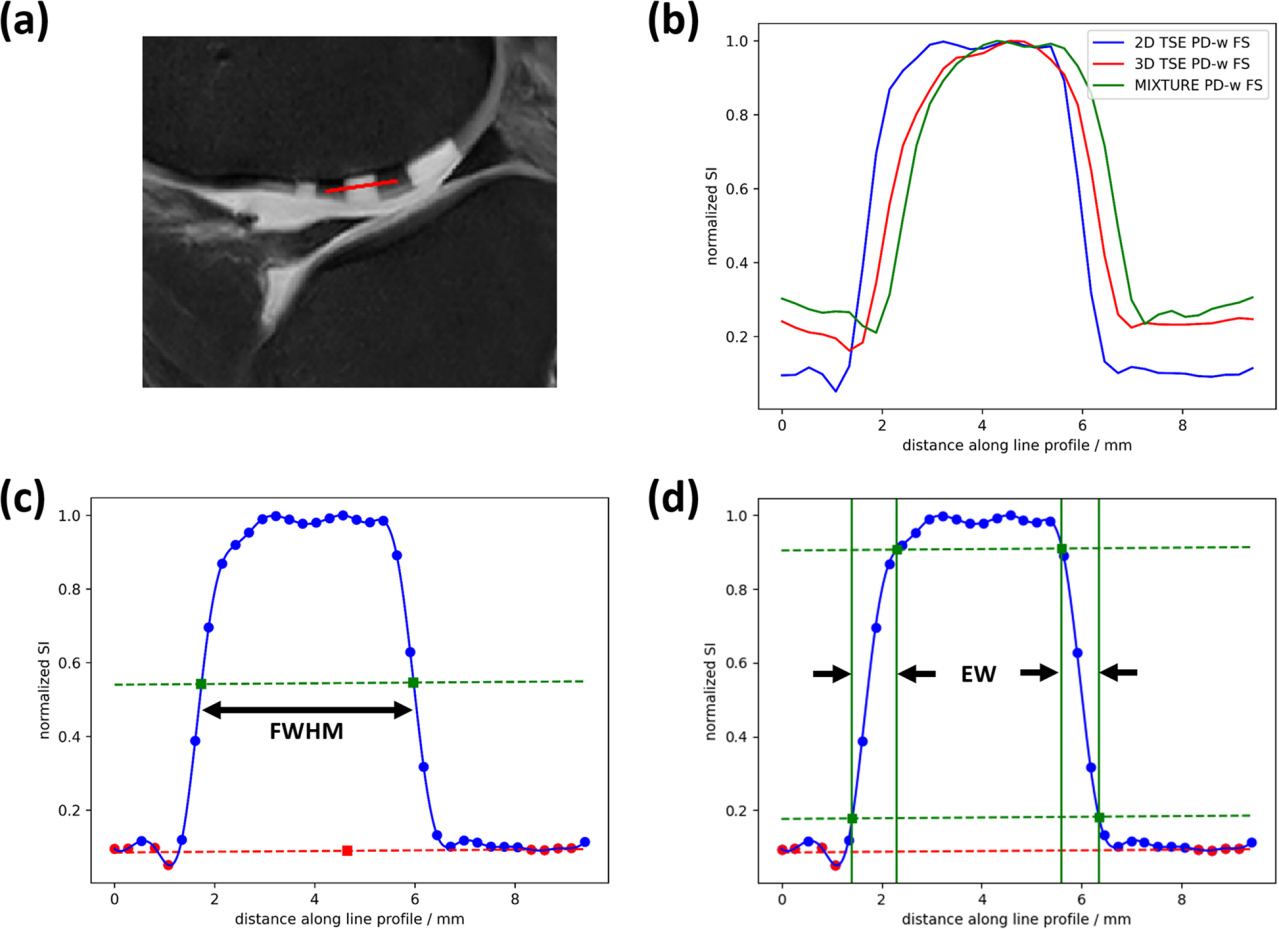
Inter-sequence comparison of cartilage defect delineability. **a** For this representative defect of 5 mm in diameter, a line was manually annotated to transect the defect and adjacent cartilage at mid-substance (red line, sagittal PD-weighted FS image). **b** For each sequence, *i.e.*, 2D TSE, 3D TSE, and MIXTURE, the line profiles (corresponding to the pixel-wise signal intensity along the red line) were extracted, normalized to the maximum signal intensity of 1 (blue circles), and used to calculate the FWHM (**c**) and the EW (**d**) as surrogate measures of defect delineability. **c** FWHM was determined by determining the half maximum (dashed green line) between the cartilage background signal intensity (dashed red line) and the maximum signal intensity and by measuring the horizontal distance between the intersecting points of the half maximum with the signal intensity profile (green dots). **d** Analogously, EW was determined by defining the horizontal distances between the 10 and 90% maximum intensity levels (green dots, dashed green lines) on both defect shoulders. *EW* Edge width, *FS* Fat-saturated, *FWHM* Full width at half maximum, *MIXTURE* Multi-interleaved x-prepared turbo spin-echo with intuitive relaxometry, *PD* Proton density, *TSE* Turbo spin-echo

Cartilage defect delineability was assessed on the PD-weighted FS sequences using line profiles manually annotated in ITK-SNAP version 3.8 [23, 24] by N.P. (pregraduation medical student, 2 years of experience in medical imaging) and visually verified by S.N. (board-certified musculoskeletal radiologist, 10 years of experience). Line profiles were placed through the defect and adjacent cartilage on the sagittal postdefect PD-weighted FS image that centrally bisected the defects (Fig. 2a). As projections of the signal intensity (SI) along their course, SI line profiles were extracted from the 2D TSE, 3D TSE, and MIXTURE PD-weighted FS images and normalized to the maximum of 1 (Fig. 2b). For every SI line profile, full width at half maximum (FWHM, Fig. 2c) and edge width (EW, Fig. 2d) were evaluated as surrogates of defect delineability. More specifically, a parallel line was defined at half maximum between the background signal level of cartilage and the maximum SI along the line profile. The horizontal distance between the intersections of this line with the SI line profile was determined as the defect’s FWHM. Similarly, two vertical lines per defect shoulder defined the 10% and 90% maximum SI. The horizontal distance between these two lines was determined as the respective defect shoulder width, and EW was calculated as the mean of both defect shoulder widths.

#### Bone texture features

**Fig. 3:**
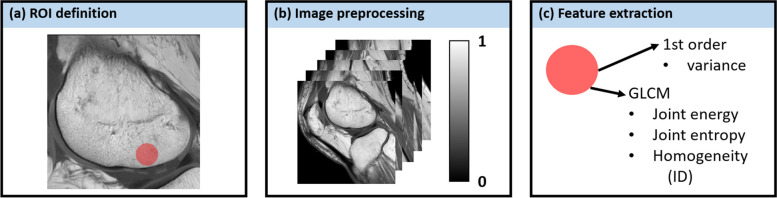
Inter-sequence comparison of bone texture. On the T1-weighted images, radiomic features were analyzed by defining a standardized circular region of interest adjacent to the subchondral lamella underneath the 5-mm defect (**a**). Image preprocessing included normalization of the signal intensity to the range of 0 to 1 (**b**). As texture features, variance as a first-order feature and joint energy, joint entropy, and homogeneity (*i.e.*, ID) as gray-level co-occurrence matrix (GLCM) features were extracted (**c**). For the computation of the GLCM, the normalized images were quantized into 200 evenly spaced bins. *GLCM* Gray-level co-occurrence matrix, *ID* Inverse difference

The bone texture on the T1-weighted sequences was quantified using radiomic features (Fig. 3). In ITK-SNAP, circular regions of interest (ROI) with a diameter of 40 pixels were defined directly adjacent to the 5-mm defect (Fig. 3a) on the same sagittal postdefect slice as above. Before computing texture features, the stacks of the 2D TSE, 3D TSE, and MIXTURE T1-weighted images were normalized between the SI values 0 and 1 (Fig. 3b). Guided by earlier studies [25], we focused on variance, (joint) energy, (joint) entropy, and inverse difference (synonymous with “homogeneity1” [PyRadiomics]) to quantify the spatial distribution of SI values, characterize the underlying bone structure, and capture what the radiologist assesses on the microstructural level (Fig. 3c). The texture features were determined using PyRadiomics [26]. Variance is a first-order feature that measures SI value spread within the ROI; high variance indicates high heterogeneity and large differences from their mean SI. Entropy, energy, and inverse difference are gray-level co-occurrence matrix features. The gray level co-occurrence matrix quantifies how often different neighboring voxel value pairs are present within the ROI. Entropy measures disorder or complexity; high entropy indicates bone tissue with a complex texture characterized by diversely varying neighboring SI values. Energy measures textural uniformity; high energy indicates many repetitions of the same neighboring SI values. Homogeneity measures local image uniformity; high homogeneity indicates more uniform gray levels. High entropy, energy, and inverse difference values indicate more randomness, homogeneous patterns, and local homogeneity [26, 27].

#### Quantitative parameter maps

N.P. segmented the femoral and tibial cartilage plates on the MIXTURE PD-weighted FS images using ITK-SNAP. The central bisecting slice through the defects (postdefect) and the corresponding original slice (predefect) were segmented. The femoral cartilage was divided into an anterior (“aF”), central (“cF”), and posterior region (“pF”) based on the outer contours of the lateral meniscus’ anterior and posterior horns. The tibial cartilage (“T”) was segmented as one region. All segmentation outlines were reviewed and adjusted by T.N. and S.N. T2 and T1ρ values were computed (predefect and postdefect) and provided as mean ± standard deviation for each region and the entire lateral femorotibial compartment. To investigate a potential interplay of cartilage morphology and relaxation times, the digital caliper of the in-house picture archiving and communication system was used to determine cartilage thickness adjacent to the 8-mm defects (postdefect images; PD-weighted FS and T1-weighted images side by side) and at the corresponding location (predefect images). The caliper’s step size was one pixel, and the caliper resolution was limited by the image resolution of 0.27 × 0.27 mm.

### Statistical analysis

Data are given as mean ± standard deviation unless differently specified. Statistical analysis was performed by N.P., T.N., and S.N. using Graph Pad Prism (v9.5.1, San Diego, CA, USA). Intersequence comparisons of FWHM, EW, and radiomic texture features were performed using repeated measures analysis of variance (ANOVA) followed by the Tukey-Kramer *post hoc* test. Predefect and postdefect T2 and T1ρ relaxation times were comparatively evaluated per region and overall using Wilcoxon matched-pairs signed-rank tests. Predefect and postdefect cartilage thickness was compared by means of a two-tailed paired *t*-test. To reduce the number of statistically significant but clinically likely irrelevant findings, the family-wise significance level was set to *∝* = 0.01. Multiplicity-adjusted *p*-values are provided.

## Results

### Study cohort

Ten knee joint specimens (age 81.1 ± 10.4 years; range 68–96 years; 9 males, 1 female) were included. Standardized cartilage defects were successfully created in all specimens.

### Qualitative evaluation

**Fig. 4:**
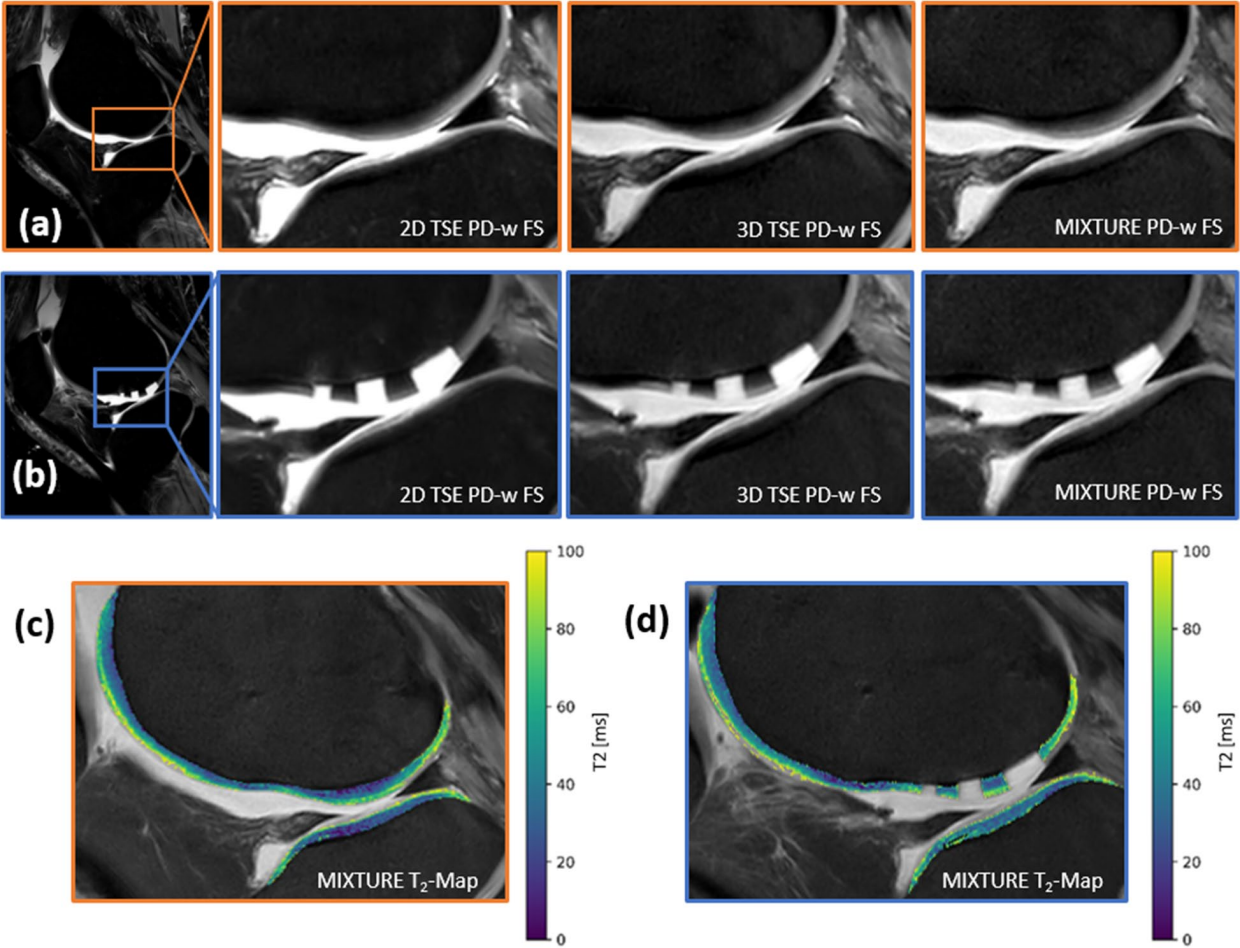
Representative PD-weighted fat-saturated images and MIXTURE T2 maps. Sagittal images before (orange frame (**a**)) and after (blue frame (**b**)) the creation of standardized cartilage defects. The slice that centrally bisected the three defects and the corresponding slice of the intact joint was selected. Cartilage defects of 3 mm, 5 mm, and 8 mm diameter (from left [anterior] to right [posterior]) are displayed. Zoomed images (indicated by the inset boxes in the leftmost images) are from left to right: the 2D TSE sequence, the 3D TSE sequence, and the MIXTURE sequence. Corresponding MIXTURE-based T2 maps before (**c**) and after (**d**) defect creation. The scale bar on the right extends from 0 to 100 ms. *MIXTURE *Multi-interleaved x-prepared turbo spin-echo with intuitive relaxometry, *PD* Proton density, *TSE* Turbo spin-echo

**Fig. 5:**
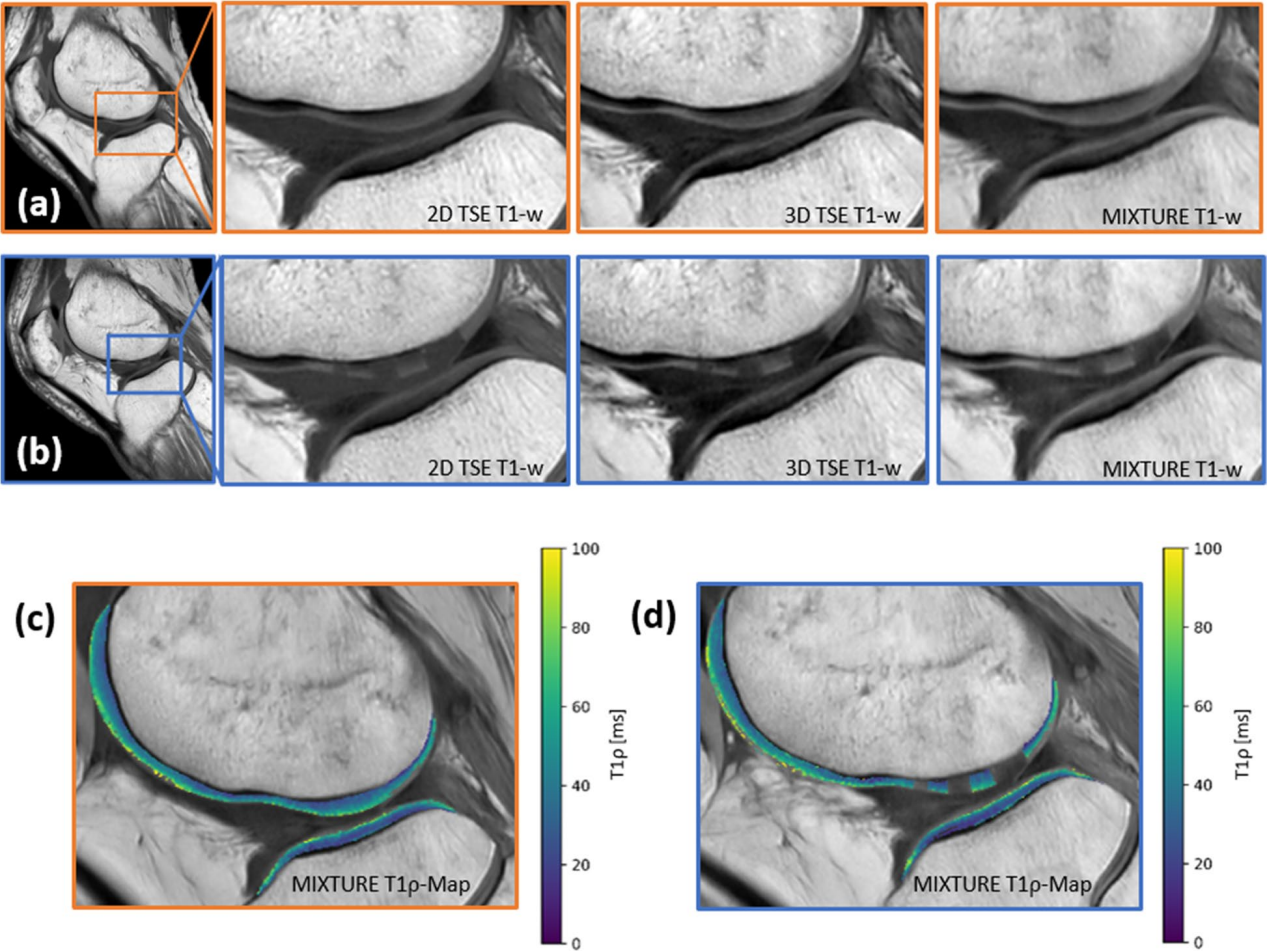
Representative T1-weighted images and MIXTURE T1ρ maps. Morphologic images (**a**, **b**) and corresponding MIXTURE-based T1ρ maps (**c**, **d**) are visualized before and after defect creation. Figure organization as in Fig. 4. *MIXTURE* Multi-interleaved x-prepared turbo spin-echo with intuitive relaxometry

In PD-weighted FS images, the cartilage defects were clearly discernable, and the cartilage tissue had the characteristic layer-wise configuration and intermediate SI in all sequences. Menisci and bone marrow appeared homogeneously dark, *i.e.*, suppressed, while intraarticular fluid was homogenously bright (Fig. 4). In the T1-weighted images, the macro- and microstructural bone texture appeared slightly less blurry in the 2D TSE sequence, particularly compared to the MIXTURE image (Fig. 5). Contrast and noise levels appeared largely similar. Regarding artifacts, we noted artificial signal hyperintensities in the menisci of two specimens on MIXTURE and 3D TSE (both PD-weighted FS) images, which were much less visible on the 2D TSE PD-weighted FS images. Their absence on the corresponding MIXTURE T2-weighted images confirmed their artificial nature (Additional file 1: Fig. S1).

### Quantitative evaluation of defect delineability

Table 2 presents the metrics of defect delineability, *i.e.*, FWHM and EW values. FWHM values were substantially lower than the nominal defect diameters but overall largely similar between the sequences. For the 5-mm defects, however, the 2D TSE sequence yielded significantly higher FWHM values than the MIXTURE sequence (*p* = 0.005). On average, EW values were lower for the 2D TSE than for the 3D TSE and MIXTURE sequences, even though statistically not significant. The latter two sequences exhibited largely similar EW values.

**Table 2:** Quantification of cartilage defect delineability

Nominal defect diameter [mm]	2D TSE FWHM [mm] EW [mm]	3D TSE FWHM [mm] EW [mm]	MIXTURE FWHM [mm] EW [mm]	*p*-value
3	2.6 ± 0.4	2.3 ± 0.7	2.6 ± 0.3	0.340
	1.0 ± 0.4	1.3 ± 0.5	1.3 ± 0.6	0.020
5	4.4 ± 0.2	4.3 ± 0.2	4.3 ± 0.2	0.004*
	1.1 ± 0.7	1.4 ± 0.4	1.4 ± 0.4	0.040
8	7.1 ± 0.5	7.0 ± 0.5	7.0 ± 0.5	0.470
	1.5 ± 0.9	1.8 ± 1.1	1.8 ± 1.0	0.450

The full widths at half maximum (FWHM) and edge widths (EW) were extracted from the line profiles of the PD-w FS images and used as surrogate parameters of defect delineability. EW was averaged over both defect shoulders. Data are presented as mean ± standard deviation [mm]. The statistical analysis was performed using repeated measures ANOVA. *p*-values are given as a function of sequence, delineability parameter (*i.e.*, FWHM and EW), and nominal defect diameter (*i.e.*, 3 mm, 5 mm, and 8 mm). Significant differences are indicated in bold type

*ANOVA* Analysis of variance, *EW* Edge width, *FS* Fat-saturated, *FWHM* Full width at half maximum, *MIXTURE* Multi-interleaved x-prepared turbo spin-echo with intuitive relaxometry, *PD* Proton density, *TSE* Turbo spin-echo

^*^The post hoc details (Tukey’s test) regarding multiplicity-adjusted *p*-values for pairwise sequence comparisons were *p* = 0.030 for 2D TSE *versus* 3D TSE, *p* = 0.005 for 2D TSE *versus* MIXTURE, and *p* = 0.910 for 3D TSE *versus* MIXTURE

### Quantitative evaluation of bone texture

**Fig. 6:**
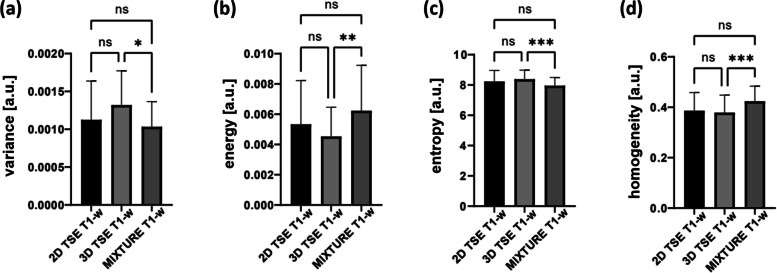
Analysis of bone texture features. Based on the radiomic feature analysis, a representative region of interest of the subchondral bone was defined and compared between the T1-weighted sequences, *i.e.*, the 2D TSE, 3D TSE, and MIXTURE sequences. Variance (**a**), energy (**b**), entropy (**c**), and homogeneity (**d**) were quantified and analyzed as measures of bone texture. Levels of statistical significance were stratified as “ns,” “*,” “**,” and “***” to indicate *p* > 0.05, 0.01 < *p* ≤ 0.05, 0.001 < *p* ≤ 0.01, and *p* ≤ 0.001, respectively. *MIXTURE* Multi-interleaved x-prepared turbo spin-echo with intuitive relaxometry, *TSE* Turbo spin-echo

Voxel SIs contained in the ROI were spread out along 43 ± 10 (2D TSE), 49 ± 9 (3D TSE), and 42 ± 9 (MIXTURE) bins, indicating a comparable spread of voxel SI distributions. The radiomic feature analysis indicated comparable bone texture feature values between the sequences (Fig. 6). When comparatively evaluating the individual features, significant differences were only found between the 3D TSE and MIXTURE sequences with significantly higher energy and homogeneity values (and significantly lower entropy values) determined for MIXTURE *versus* 3D TSE.

### Quantitative parameter maps

We observed increased T2 and T1ρ relaxation times after defect creation for all studied regions (Table 3). These increases were mainly non-significant except for T2 in the central femur, where the defects were located (predefect, 51 ± 4 ms; postdefect, 56 ± 4 ms; *p* = 0.002), and for T1ρ when considering all regions together (predefect, 40 ± 4 ms; postdefect, 43 ± 4 ms; *p* = 0.004). The average cartilage thickness increased from 2.9 ± 0.5 mm (predefect) to 3.1 ± 0.5 mm (postdefect) (*p* = 0.06).

**Table 3:** Quantification of cartilage composition and ultrastructure

Region	T2_pre_ [ms]	T2_post_ [ms]	*p*-value	T1ρ_pre_ [ms]	T1ρ_post_ [ms]	*p*-value
Anterior femur	48 ± 3	49 ± 3	0.43	43 ± 5	46 ± 3	0.040
Central femur	51 ± 4	56 ± 4	0.002*	41 ± 5	43 ± 5	0.190
Posterior femur	64 ± 8	69 ± 9	0.04	34 ± 9	39 ± 8	0.020
Tibia	41 ± 4	45 ± 4	0.05	36 ± 4	40 ± 5	0.010
All regions	48 ± 2	51 ± 2	0.03	40 ± 4	43 ± 4	0.004*

T2 and T1ρ relaxation times (mean ± standard deviation [ms]) of the segmented cartilage of the central lateral femorotibial compartment before and after defect creation in ten knee joint specimens. The regional assessment included three femoral and one tibial region. Predefect and postdefect relaxation times were compared using the Wilcoxon matched-pairs signed rank test, and multiplicity-adjusted *p*-values were determined

*Post* After defect creation, *Pre* Before defect creation

^*^Significant difference

## Discussion

Our study evaluated the image quality of MIXTURE PD-weighted FS and T1-weighted sequences relative to corresponding 2D and 3D TSE reference sequences. Focusing on the delineability of cartilage defects and quantitative bone texture features, we found that MIXTURE sequences were largely equivalent regarding image contrast, morphologic correspondence and coherence, and quantitative features. Simultaneously, MIXTURE sequences provided quantitative T2 or T1ρ maps with little additional scan time. Thereby, MIXTURE sequences could increase the diagnostic information of routine scan protocols beyond mere morphology and may complement (or in parts even replace) current knee MRI protocols.

The primary advantage of MIXTURE sequences is their TSE-derived image contrast. Since their introduction to the clinic in the early 1990s, TSE sequences have been considered the standard for knee MRI; thus, radiologists are used to these images, and the American College of Radiology even formally recommends their usage [28]. MIXTURE sequences obviate the need for radiologists to familiarize themselves with other contrasts. Additionally, the sequence architecture is flexible and may be adjusted to other TSE-based weightings with or without fat saturation. A broad spectrum of sequence combinations can thus be efficiently acquired at each institution’s discretion.

Specifically, we evaluated a PD-weighted FS sequence with T2 maps and a T1-weighted sequence with T1ρ maps acquired with 43 slices across the joint in 5 and 6.5 min, respectively. Previously, Kijowski et al. [3] highlighted the clinical potential of adding T2 maps to the routine protocol. Even though the diagnostic benefit of T1ρ maps remains unclear, adding more quantitative images to the morphologic standard images seems well-justified. MIXTURE needs at least two morphologic images, which require more acquisition time than a single image.

The MIXTURE PD-weighted FS sequence depicted the cartilage defects with a level of contrast and sharpness similar to the reference sequences. By trend, EW and FWHM values of the 2D TSE sequences were lower and closer to the nominal defect diameters, respectively, than those of the corresponding MIXTURE and 3D TSE sequences. This finding indicates slightly less clear defect delineability of the latter sequences and may be due to the higher echo train lengths [29, 30] or the choice of the refocusing pattern that, besides affecting image contrast and SNR, also influences image blurring [31].

Increased blurring, likely secondary to the choice of the refocusing pattern, was observed for the MIXTURE T1-weighted images and confirmed by the radiomic analysis of bone texture. Bone texture was significantly more homogeneous in the MIXTURE T1-weighted sequence, which may translate into a loss of microtextural detail with as yet unknown clinical relevance.

When designing the study, we aimed to compare cartilage and bone texture voxel-wise. To this end, we matched the image resolutions of all sequences, both in plane and through plane. Yet, this approach precluded the possibility of performing multiplanar reconstructions, an inherent feature of isotropic 3D sequences, which is a prerequisite for precise tissue segmentation (of cartilage and meniscus) for the analysis of morphometry and relaxivity [32, 33].

Quantitative analyses indicated increased postdefect relaxation times. Alongside increased T2 and T1 ρ relaxation times, we observed cartilage thickness increases when comparing predefect and postdefect images. Even though the limited number of specimens and the caliper-related inaccuracies in thickness measurements need to be acknowledged, the changes in cartilage morphology and relaxivity are likely due to tissue swelling secondary to surgical handling, extended exposure to unphysiological conditions, and potentially altered tonicity.

While the exact compositional and structural correlates of prolonged T1ρ and T2 relaxation times remain unknown, literature evidence suggests that cartilage hydration is likely dominant [34, 35]. Surprisingly, we observed higher T2 than T1ρ relaxation times in the cartilage. In biological tissues, T1ρ relaxation times should be longer than T2 relaxation times because the spin-lock pulse forces the spins to precess about a direction different from the main magnetic field *B*_0_, thereby slowing T2 relaxation [36]. Shorter repetition times (as present in the MIXTURE sequence) may have led to T1ρ underestimation [37]: if the repetition time is too short, it may not allow for complete T1ρ relaxation and decrease T1ρ relaxation times. Other factors worth considering are the applied radiofrequency pulse for the T1ρ preparation, the *B*_1_ inhomogeneity, and the magic angle effect [38]. Future phantom studies are needed to assess the accuracy and validity of MIXTURE-based relaxivity measurements *versus* reference measurements, *e.g.*, multi-echo spin-echo sequences (for T2 quantification) and gradient-echo sequences (for T1ρ quantification) [39].

Our study has limitations. First, the *in situ* defect model using human cadaveric knee joints only approximates the actual *in vivo* situation. However, the model effectively excludes intersequence motion (and other artifacts such as arterial pulsations) and helps realize reproducible and standardized experimental conditions for voxel-wise comparisons. Regarding clinical translation, this model is inherently limited. Second, the number of specimens was small, and the study provided, by design, a focused proof of concept. Further diagnostic aspects relating to particular knee joint conditions require larger sample sizes and, ideally, assessment in the clinical routine.

In conclusion, combined morphologic and quantitative MRI sequences, such as the versatile MIXTURE platform, increase scanning efficiency and diagnostic utility by providing familiar contrasts and delivering additional quantitative information about joint cartilage defects in human cadaveric specimens. In a basic research context, MIXTURE sequences demonstrated excellent delineability of cartilage defects and visualization of bone texture on par with the corresponding reference sequences. Once corroborated by larger clinical studies, MIXTURE may be a promising sequence platform for comprehensive and time-efficient joint imaging.

### Supplementary Information

**Additional file 1:** **Fig. S1.** Intra-meniscal artifacts as a function of sequence. In the posterior horn of the lateral meniscus, the hyperintense signal discernable in the PD-weighted FS images, *i.e.*, 3D TSE, MIXTURE, and (much less pronounced) in 2D TSE, was determined to be artificial when assessed against the corresponding MIXTURE T2-weighted image (green arrows). Corresponding slices in the sagittal orientation. Abbreviations: FS fat saturated, MIXTURE Multi-Interleaved X-prepared Turbo-Spin Echo with IntUitive Relaxometry, TSE turbo spin echo, PD proton density.(PDF 149 KB)

## Data Availability

The datasets used and/or analyzed during the current study are available from the corresponding author (TL) upon reasonable request.

## Abbreviations

2D: Two-dimensional
3D: Three-dimensional
ANOVA: Analysis of variance
EW: Edge width
FS: Fat-saturated
FWHM: Full width at half maximum
MIXTURE: Multi-interleaved x-prepared turbo spin-echo with intuitive relaxometry
MRI: Magnetic resonance imaging
PD: Proton density
ROI: Region of interest
SI: Signal intensity
SPAIR: Spectral attenuated inversion-recovery
TSE: Turbo spin-echo
