# Supplementary material for: Time-efficient combined morphologic and quantitative joint MRI: an in situ study of standardized knee cartilage defects in human cadaveric specimens
Source: Eur Radiol Exp. 2024 Jun 5;8:66. doi: 10.1186/s41747-024-00462-0 (PMC11150352; doi:10.1186/s41747-024-00462-0)
Supplement: Supplementary file 1 — Additional file 1: Fig. S1. Intra-meniscal artifacts as a function of sequence. In the posterior horn of the lateral meniscus, the hyperintense signal discernable in the PD-weighted FS images, i.e., 3D TSE, MIXTURE, and (much less pronounced) in 2D TSE, was determined to be artificial when assessed against the corresponding MIXTURE T2-weighted image (green arrows). Corresponding slices in the sagittal orientation. Abbreviations: FS fat saturated, MIXTURE Multi-Interleaved X-prepared Turbo-Spin Echo with IntUitive Relaxometry, TSE turbo spin echo, PD proton density.(PDF 149 KB) [file 41747_2024_462_MOESM1_ESM.docx]

# Supplementary material

## Supplementary Figure S1

| 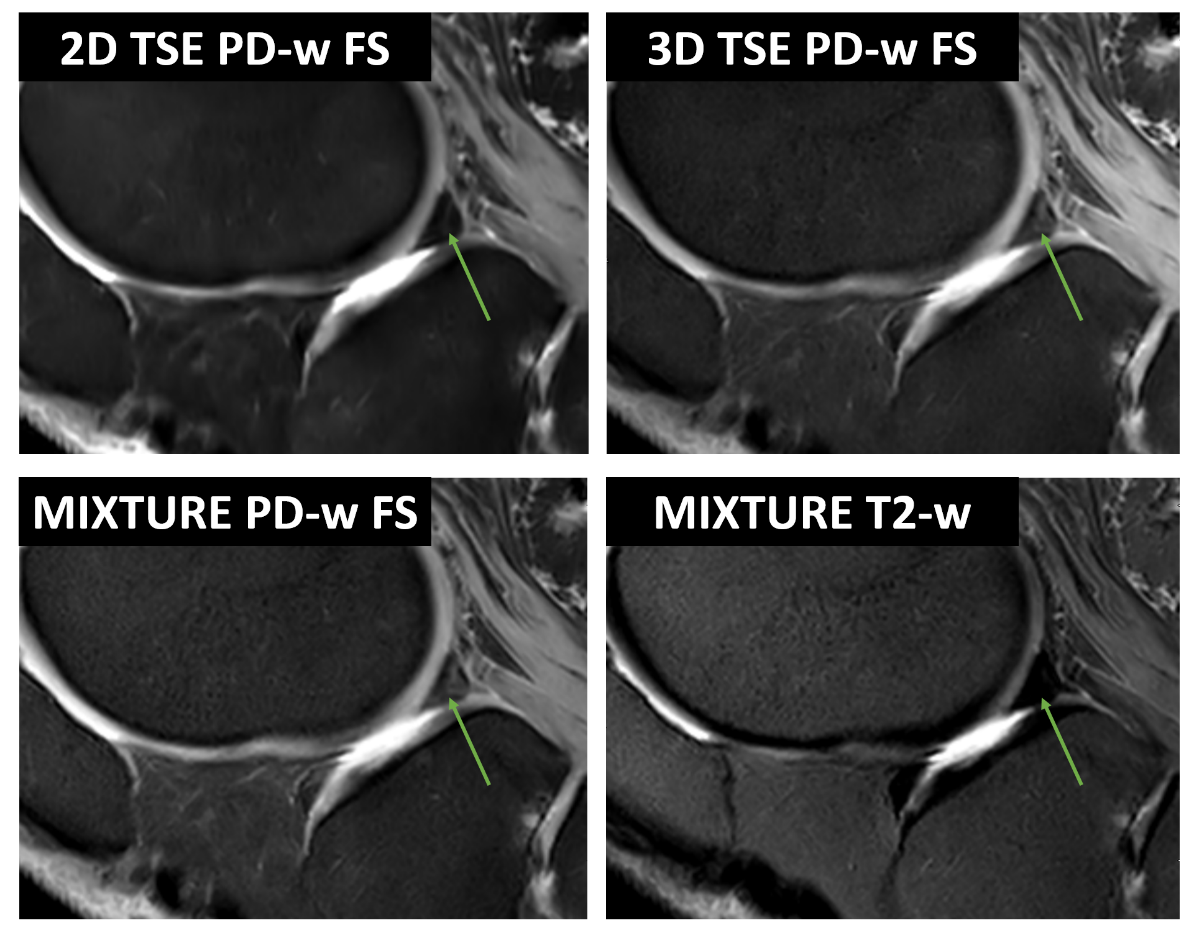 |
| --- |
| **Supplementary Figure 1: Intra-meniscal artifacts as a function of sequence.** In the posterior horn of the lateral meniscus, the hyperintense signal discernable in the PD-weighted FS images, i.e., 3D TSE, MIXTURE, and (much less pronounced) in 2D TSE, was determined to be artificial when assessed against the corresponding MIXTURE T2-weighted image (green arrows). Corresponding slices in the sagittal orientation.  Abbreviations: FS fat saturated, MIXTURE Multi-Interleaved X-prepared Turbo-Spin Echo with IntUitive Relaxometry, TSE turbo spin echo, PD proton density |
